# Proteomic Analysis of Mucopolysaccharidosis IIIB Mouse Brain

**DOI:** 10.3390/biom10030355

**Published:** 2020-02-26

**Authors:** Valeria De Pasquale, Michele Costanzo, Rosa Anna Siciliano, Maria Fiorella Mazzeo, Valeria Pistorio, Laura Bianchi, Emanuela Marchese, Margherita Ruoppolo, Luigi Michele Pavone, Marianna Caterino

**Affiliations:** 1 Department of Molecular Medicine and Medical Biotechnology, School of Medicine, University of Naples Federico II, 80131 Naples, Italy; valeria.depasquale@unina.it (V.D.P.); michele.costanzo@unina.it (M.C.); valeria.pistorio@unina.it (V.P.); margherita.ruoppolo@unina.it (M.R.); marianna.caterino@unina.it (M.C.); 2 CEINGE-Biotecnologie Avanzate scarl, 80145 Naples, Italy; emanuela.marchese89@gmail.com; 3 Institute of Food Sciences, CNR, 83100 Avellino, Italy; rosa.siciliano@isa.cnr.it (R.A.S.); fiorella.mazzeo@isa.cnr.it (M.F.M.); 4 Laboratory of Functional Proteomics, Department of Life Sciences, University of Siena, 53100 Siena, Italy; bianchi12@unisi.it; 5 Department of Mental Health and Preventive Medicine, University of Campania Luigi Vanvitelli, 80138 Naples, Italy

**Keywords:** mucopolysaccharidosis IIIB, quantitative proteomics, NAGLU, lysosomes

## Abstract

Mucopolysaccharidosis IIIB (MPS IIIB) is an inherited metabolic disease due to deficiency of α-N-Acetylglucosaminidase (NAGLU) enzyme with subsequent storage of undegraded heparan sulfate (HS). The main clinical manifestations of the disease are profound intellectual disability and neurodegeneration. A label-free quantitative proteomic approach was applied to compare the proteome profile of brains from MPS IIIB and control mice to identify altered neuropathological pathways of MPS IIIB. Proteins were identified through a bottom up analysis and 130 were significantly under-represented and 74 over-represented in MPS IIIB mouse brains compared to wild type (WT). Multiple bioinformatic analyses allowed to identify three major clusters of the differentially abundant proteins: proteins involved in cytoskeletal regulation, synaptic vesicle trafficking, and energy metabolism. The proteome profile of NAGLU^−/−^ mouse brain could pave the way for further studies aimed at identifying novel therapeutic targets for the MPS IIIB. Data are available via ProteomeXchange with the identifier PXD017363.

## 1. Introduction

Mucopolysaccharidosis type IIIB (MPS IIIB) is an inherited metabolic disease caused by the deficiency of the enzyme α-N-Acetylglucosaminidase (NAGLU, EC: 3.2.1.50) required for the degradation of the glycosaminoglycan (GAG) heparan sulfate (HS) [1,2]. The undigested HS accumulates in different tissues leading to progressive cellular damage and organ dysfunction, with the central nervous system (CNS) being the primary site of the pathology [3,4,5,6,7]. The CNS pathology in MPS IIIB patients comprises hydrocephalus, behavioral disorders, sleep disturbances, vision and progressive hearing loss, learning delay, and intellectual disability [8,9]. Although different pathophysiological mechanisms have been investigated both in the brain of MPS IIIB patients and in animal models of the disease [9], the etiology of the neurological dysfunction in MPS IIIB is still unclear. The characteristic pathological changes include white matter abnormalities, cortical and corpus callosum atrophy [10,11], cerebellar atrophy with loss of Purkinje cells [12,13], retinal epithelium pigmentation loss, and photoreceptor degeneration [14]. Accumulation of specific HS glycoforms in neurons and glial cells in the brain of MPS IIIB mouse model has been associated with increased expression of HS biosynthetic enzymes, may contributing to the neuropathology of MPS IIIB by exacerbating the lysosomal HS storage. [15,16]. Secondary accumulation of gangliosides in the brain of patients and MPS IIIB mice has also been documented [17,18]. The molecular mechanisms underlying the neuropathology in MPS IIIB appear to imply a complex interplay between the activation of glial cells, alterations of the oxidative status, as well as neuroinflammation [19,20,21]. However, there are still many issues to be elucidated.

In this study, a comparative analysis of the proteome profiles of MPS IIIB and wild type (WT) mouse brains was performed using a quantitative proteomic approach [22,23,24,25], which allowed us to identify 204 proteins that were significantly differentially expressed in the brain of MPS IIIB versus WT mice. Multiple bioinformatic analyses using PANTHER [26], REACTOME, [27] STRING [28] and MetaCore [29] databases allowed a functional classification of the detected proteins and highlighted the biological pathways perturbed in MPS IIIB brains. These results might provide useful tools for further studies aimed to identify molecular targets for therapies.

## 2. Material and Methods

### 2.1. Animal Description

MPS IIIB knockout mice (NAGLU^−/−^) available to us were generated by Prof. Elizabeth Neufeld, University of California, Los Angeles (UCLA), by insertion of neomycin resistance gene into exon 6 of NALGU gene on the C57/BL6 background [30]. NAGLU^−/−^ and WT mice were genotyped by PCR [31]. Mice were housed with no more than four per cage, maintained under identical conditions of temperature (21 ± 1 °C), humidity (60% ± 5%) and light/dark cycle, and had free access to normal mouse chow. All animal experiments were in compliance with the ARRIVE (Animal Research: Reporting of in vivo Experiments) guidelines and were carried out in accordance with the EU Directive 2010/63/EU for animal experiments. All mouse care and handling procedures were approved by the rules of the Institutional Animal Care and Use Committee (IACUC) of the Centre of Biotechnologies A.O.R.N. “Antonio Cardarelli” (Naples 80131, Italy).

### 2.2. Sample Collection and Preparation

Whole mouse brains were collected from five MPS IIIB and five WT male mice (8-month old) for further analysis [32,33]. Brain tissues were lysed in ice-cold lysis buffer containing 7 M urea, 2 M thiourea, 4% cholamidopropyl dimethylammonio 1 propanesulfonate (CHAPS), 30 mM Tris-HCl pH = 7.8 supplemented with protease inhibitor cocktail (Roche, Indianapolis, IN, USA) [34]. Mechanical disruption of tissues was performed on ice using 2 mL Dounce homogenizer applying ten strokes per brain sample [35]. Lysed tissues were centrifuged for 20 min at 13,000 rpm; supernatants were collected and protein concentration was quantified by Bradford assay using Bio-Rad Protein Assay Dye Reagent Concentrate (Hercules, CA, USA). Protein extract aliquots (50 μg) from the five biological replicates for each condition (NAGLU^−/−^ and WT) were fractionated on 10% SDS–PAGE. The resolved proteins were stained using Gel Code Blue Stain Reagent (Thermo Fisher Scientific, Waltham, MA, USA) and each gel lane was divided into five pieces and hydrolyzed by in situ trypsin digestion as previously described [36,37,38].

### 2.3. LC–MS/MS Analysis

Peptide mixtures were dissolved in 0.2% HCOOH and analyzed by LC–MS/MS using a Q-Exactive^TM^ mass spectrometer (Thermo Scientific, Bremen, Germany) coupled with an UltiMate 3000 RSLC nanoLC system (Thermo Scientific). The peptide mixture was desalted by a precolumn (Acclaim PepMap C18, 300 μm × 5 mm nanoViper, 5 μm, 100 Å, Thermo Scientific), in 0.05% formic acid and 2% acetonitrile. Finally, the peptide mixture was fractionated on a reverse phase capillary column (Acclaim Easy Spray PepMap RSLC C18, 75 μm × 15cm nanoViper, 3μm, 100 Å, Thermo Scientific) and eluted by a nonlinear gradient: 4% B for 5 min, from 4% to 40% B in 45 min and from 40% to 90% B in 1 min at flow rate of 300 nL/min. (Eluent A, 0.1% formic acid; Eluent B, 80% acetonitrile, 0.08% formic acid). MS analysis was setup as follows: data dependent full MS/ddMS2; ten most intense precursor ions fragmentation; 30 sec dynamic exclusion; MS resolution 70,000; MS/MS resolution 17,500 [39,40].

### 2.4. Protein Identification and Data Processing

The identification of proteins in brain samples from WT and MPS IIIB mice was performed using the platform Thermo Proteome Discoverer™ (version 1.3.0.339, Thermo Scientific, Bremen, Germany), combined with the use of the SEQUEST HT Search Engine server (University of Washington, Seattle, WA, USA).

The peak lists were processed according to the follow parameters: (I) Spectrum Selector. Minimum Precursor Mass: 350 Da, Maximum Precursor Mass: 5000 Da, Minimum Peak Count: 1; (II) SEQUEST HT: 1. Input Data. Protein Database: Swiss-Prot, Enzyme: Trypsin, Maximum Missed Cleavage Sites: 2, Instrument: Electrospray Ionization Fourier Transform Ion Cyclotron Resonance Mass Spectrometer (ESI FT-ICR MS), Taxonomy: *Mus Musculus*. 2. Tolerances. Precursor Mass Tolerance: 5 ppm, Fragment Mass Tolerance: 0.8 Da. 3. Dynamic Modification. Methionine Oxidation, N-terminal Glutamine cyclization to Pyroglutamic Acid, N-terminal protein Acetylation. 4. Static modification: Cysteine Carboamidomethylation; (III) Target Decoy/PSM validator: 1. Maximum Delta Cn: 0.05; 2. Target false discovery rate (FDR) (strict): 0.01; 3. Target FDR (relaxed): 0.05. Proteins identified by a minimum of three peptides along the replicates were accepted. The dataset has been deposited to ProteomeXchange via the PRIDE database (PXD017363).

### 2.5. Quantitative Label-Free Proteomic Analysis

The relative abundances of proteins within the proteomic datasets were compared between the two groups, NAGLU^−/−^ and WT murine brains, according to the spectral counting approach [34]. The quantitative analysis was performed by calculating the Normalized Spectral Abundance Factor (NSAF) as the number of spectral counts (SpCs) of each protein divided by protein length (SAF) and normalized for the sum of SAFs in a given lane. A Student’s *t* test was used to select proteins showing significant changes between the analyzed datasets, resulting in two-tailed *p* values. The value of *p* < 0.05 was considered to be statistically significant. In order to measure the relative abundance for each identified protein and significantly represented into the two datasets, Fold_NSAF_ was calculated as log2 (NSAF1/NSAF2), where NSAF1 was referred to the mean of NAGLU^−/−^ samples NSAF, and NSAF2 to the mean of WT samples, respectively. Fold_NSAF_ was reported as abundance index [38].

### 2.6. Western Blot Analysis

The total protein extract (50 µg) from NAGLU^−/−^ and WT murine brains was analyzed by Western blotting with the rabbit monoclonal antibody anti-Gfap (ab-68428, Abcam). Mouse anti-β-actin monoclonal antibody (G043) from Abm was used to ensure equal loading of proteins in all lanes.

### 2.7. Bioinformatic Analysis

To investigate the molecular pathways influenced by NAGLU depletion in murine brain tissues, the identified proteomic dataset was analyzed by using the PANTHER (Protein ANalysis THrough Evolutionary Relationship) database available online at http://www.pantherdb.org [41,42,43] and the REACTOME database available online at https://www.reactome.org. Results of the PANTHER analyses for the biological process enrichment and pathway enrichment were expressed as percentage of protein listed in each category. The deregulated protein dataset was also processed using STRING (Search Tool for the Retrieval of Interacting Genes) functional protein association networks (http://string-db.org/) in order to identify protein networks linked to the differentially expressed proteins. The identified networks were evaluated by a significant score as negative logarithm of the *p*-value. The differentially expressed proteome dataset (*n* = 204 proteins) was further analyzed by the MetaCore™ resource (Clarivate Analytics, London, UK) in order to investigate protein functional interconnections. To facilitate the software processing, protein differences were processed using their corresponding EntrezGene IDs. The EntrezGene ID list was imported into MetaCore and processed for functional enrichment by “diseases by biomarkers” and “process networks” ontologies using the Functional Ontology Enrichment tool. While the “diseases by biomarkers” enrichment analysis allows for clustering proteins that were annotated as statistically significant biomarkers in characterized pathologies, the “process networks” analysis visualizes the involvement of experimental proteins in biochemical and molecular processes of biological systems. The differentially abundant proteins that characterize NAGLU^−/−^ mouse brains were also investigated by using the MetaCore Network Building tool software that functionally crosslinks proteins under processing and builds protein networks. The Shortest Path algorithm was selected to highlight tight functional correlation existing among experimental proteins. It actually allows for inclusion in the same net only those proteins that directly interact or that are functionally correlated by a further factor not present in the processed protein list, but that is known to act as a molecular functional bridge between them. The relevant obtained pathway maps are indeed prioritized according to their statistical significance (*p* ≤ 0.001) and graphically visualized as nodes (proteins) and edges (interconnections among proteins). All annotations used by the MetaCore tools are from an in-house database, periodically updated and built by extrapolating information from highly reliable scientific sources.

## 3. Results

### 3.1. SDS–PAGE and Protein Identification in Brain Samples from MPS IIIB and WT Mice

**Figure 1:**
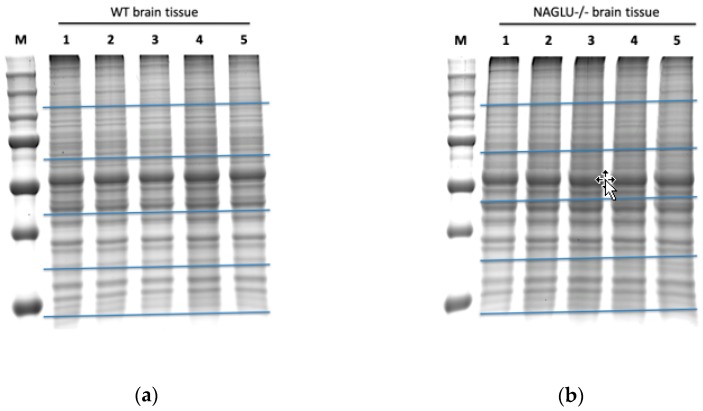
SDS–PAGE of proteins from murine WT (wild type) and NAGLU^−/−^ (α-N-Acetylglucosaminidase) brain tissues. Proteins from five murine WT brains (**a**) and five murine NAGLU^−/−^ brains (**b**) were resolved on a 10% SDS–polyacrylamide gel and stained by a gel code blue stain reagent. Each gel lane was fractionated at the level of the blue horizontal bars in order to obtain five gel bands per sample.

Brain samples from five MPS IIIB and five WT mice were collected in order to identify differentially expressed proteins through a label-free proteomic analysis and their protein extracts were independently fractionated by SDS–PAGE (Figure 1). Each gel lane was divided into five pieces and each piece was hydrolyzed by in situ trypsin digestion.

Peptide mixtures from each of the five biological replicates were analyzed two times by LC–MS/MS with a Q-Exactive mass spectrometer coupled with a nanoLC system. From the two MS analyses, we obtained ten protein datasets for NAGLU^−/−^ and ten protein datasets for WT mice. The MS details of protein identifications are listed in Table S1.

Proteins identified by a minimum of two peptides in the 70% (7/10) of the analyzed replicates were included in the dataset that underwent further quantitative analysis.

### 3.2. Quantitative Analysis of Differentially Expressed Proteins in Brain Samples from MPS IIIB and WT Mice

The spectral count abundance parameter NSAF was calculated for each protein. Its variability was evaluated within the technical replicates into the same biological replicate by linear regression of the correlation (Supplemental Figure S1). The R-squared values for WT 1, WT 2, WT 3, WT 4, WT 5, and NAGLU^−/−^ 1, NAGLU^−/−^ 2, NAGLU^−/−^ 3, NAGLU^−/−^ 4, NAGLU^−/−^ 5, were 0.983, 0.994, 0.993, 0.993, 0.995, and 0.989, 0.986, 0.983, 0.990, 0.991, respectively. These results show that the data have a high quantitative reproducibility. Normal distribution of the NSAF parameter both in WT and NAGLU^−/−^ dataset was verified (Supplemental Figure S2A). Moreover, similarities in the WT and NAGLU^−/−^ proteomes was evaluated by using multivariate analysis PCA (Supplemental Figure S2B). No outliers are found in plots of PCA scores. Finally, the overall sample correlation matrix is shown in Supplemental Figure S2C.

Protein relative abundance was then calculated by a spectral counting approach using the FoldNSAF, and Table 1 shows the list of these significant differentially abundant proteins. Table 1 includes the Swiss-Prot accession code, gene name, protein description, Fold_NSAF_ value, *p* value, and subcellular localization (Uniport database) for each protein. The volcano plot analysis of the global proteome comparison between NAGLU^−/−^ and WT is shown in Supplemental Figure S3.

Overall, the NAGLU^−/−^ brain proteome dataset showed 130 under-represented and 74 over-represented proteins compared to WT mice. For technical validation of the label-free quantitative proteomic analysis, Gfap (P03995) differential abundance was proved by Western blotting analysis using an independent set of brain sample from NAGLU^−/−^ and WT mice (Figure S4).

### 3.3. Bioinformatic Analysis of Differentially Abundant Proteins

**Figure 2:**
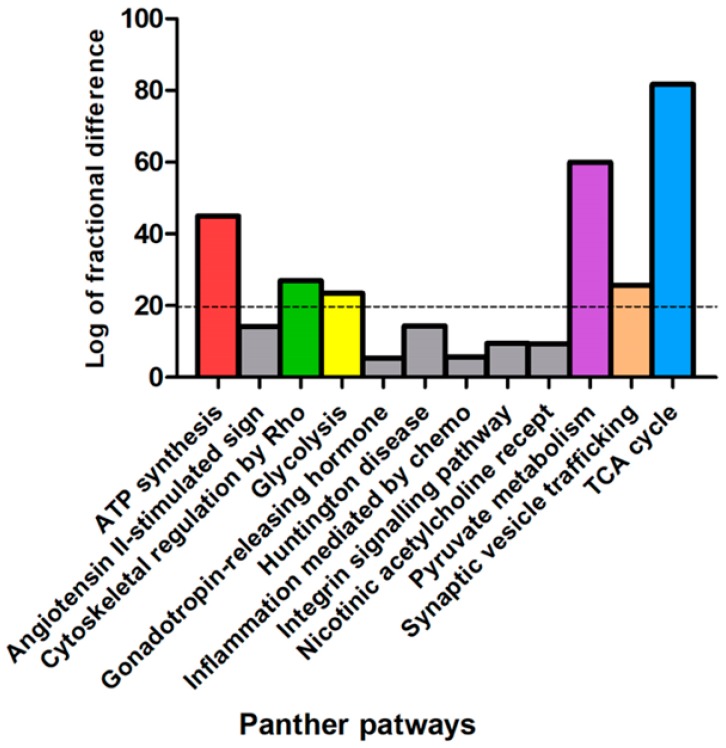
PANTHER pathways classification of murine NAGLU^−/−^ brain tissue proteome. The dysregulated proteins in NAGLU^−/−^ brain versus murine WT brain proteomes were clustered according to their cellular pathways using the Protein Analysis Through Evolutionary Relationship (PANTHER) software. The PANTHER pathways graphical enrichment (*y*-axis) was associated to each cellular pathway (*x* axis). The PANTHER cellular pathways were listed according to enriched values, expressed as the log of fractional difference (observed vs expected): (number of genes for the category – number of genes expected) / (number of genes expected).

**Figure 3:**
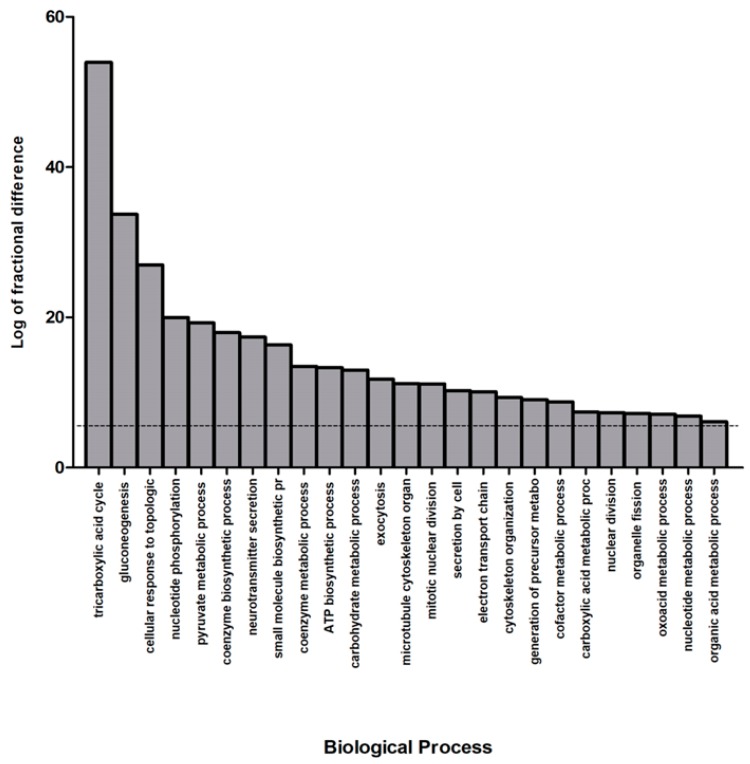
Biological process classification of murine NAGLU^−/−^ brain tissue proteome. The differentially expressed proteins in NAGLU^−/−^ brain versus murine WT brain proteomes were clustered according to their gene ontology (GO) biological process using PANTHER software. The most significant biological processes are listed according to their enriched values, expressed as the log of fractional difference (observed vs. expected): (number of genes for the category – number of genes expected) / (number of genes expected).

In order to elucidate the biological implications of the differentially abundant proteins in NAGLU^−/−^ brain tissue, the whole proteome profile containing the upregulated and downregulated proteins were analyzed by the PANTHER database, which allows classification and identification of protein functions. The PANTHER enrichment analysis allowed for clustering differentially abundant proteins with respect to cellular pathways (Figure 2 and Table S2) and biological processes (Figure 3 and Table S3).

Figure 2 shows the graphical enrichment of cellular pathways in the deregulated dataset, expressed as the log of fractional difference (observed vs. expected): (number of genes for the category – number of genes expected) / (number of genes expected). The most significant and enriched processes (enrichment threshold > 20) are shown in Figure 2 as “TCA cycle” (81.7, *p* 1.99 × 10^−8^), “Pyruvate metabolism” (59.9, *p* 1.54 × 10^−6^), “ATP Synthesis” (44.9, *p* 1.33 × 10^−3^), “Cytoskeletal regulation by Rho GTPase” (27.0, *p* 1.19 × 10^−13^), “Synaptic vesicle trafficking” (25.7, *p* 2.84 × 10^−5^), and “Glycolysis” (23.4, *p* 3.91 × 10^−4^) (Table S2).

Figure 3 and Table S3 show the most significant biological processes in our dataset. Among the significant categories the most interesting processes are the “tricarboxylic acid cycle” (53.9% enriched value) and “gluconeogenesis” (33.7% enriched value). Interestingly, a variety of pathways resulted deregulated in the MPS IIIB mouse brains, indicating the complexity of the pathogenesis for the disease.

In order to obtain additional information from our dataset, the NAGLU^−/−^ differentially abundant proteins were also analyzed using REACTOME and the MetaCore functional ontology enrichment tools.

The REACTOME clustering (Table 2) shows three major significant clusters: “Microtubule-dependent trafficking of connexons from Golgi to the plasma membrane” (R-MMU-190840; *p* 1.45 × 10^−11^), “Transport of connexons to the plasma membrane” (R-MMU-190872, *p* 2.12 × 10^−11^) and “Citric acid cycle (TCA cycle)” (R-MMU-71403, *p* 3.68 × 10^−12^). REACTOME and PANTHER analyses indeed suggest that altered protein trafficking and cellular metabolism play a crucial role in the neuropathology of MPS IIIB disease.

**Figure 4:**
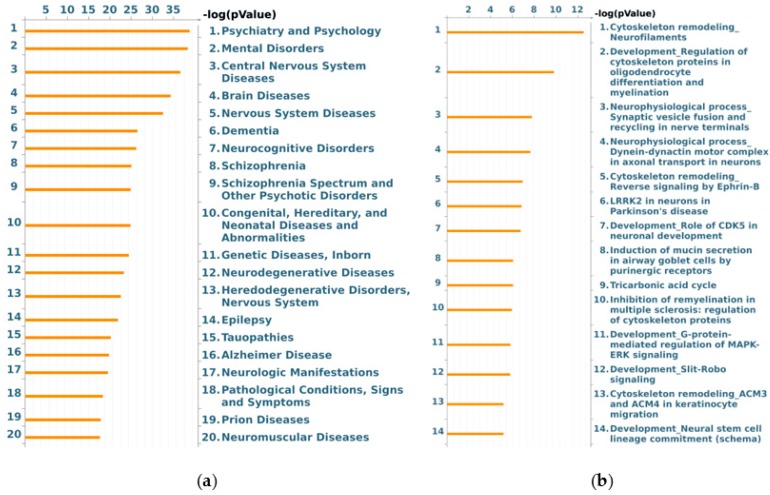
MetaCore “diseases by biomarkers” (**a**) and “process networks” (**b**) enrichment analysis of murine NAGLU^−/−^ brain tissues proteome. The MetaCore enriched analysis provides a statistically supported list of (a) diseases in which identified proteins have been previously described as biomarkers; and (b) process networks more represented in identified proteins dataset.

On the other hand, the MetaCore functional ontology enrichment tool was applied to map identified proteins into two MetaCore ontologies: “diseases by biomarkers” and “process networks”. The 20 most significant enriched terms are reported in Figure 4, where the –log(pValue)s of the mapping of the experimental protein set to the ontology terms “diseases by biomarkers” (Figure 4a; FDR ≤ 2.5 × 10^−11^) and “process networks” (Figure 4b; FDR ≤ 2.8 × 10^−3^) are represented by histograms. The former enrichment highlighted the tight correlation existing between identified differentially abundant proteins and CNS affections. As expected, a number of differences were annotated as biomarkers in (i) CNS diseases, e.g., in some heredodegenerative disorders, prion diseases, tauopathies, and Alzheimer disease; (ii) dementia, epilepsy, and neurocognitive impairments; and iii) mental disorders, as schizophrenia and other psychotic disorders (Table S4).

According to “process networks” ontologies, and in line with PANTHER and REACTOME results, cytoskeleton dynamics were suggested as the most representative cellular process affected in NAGLU^−/−^ mouse brains. Spanning from microtubule and microfilaments to intermediate filaments, the three main structural and functional components of cytoskeleton seem actually altered by MPS IIIB. Related to cytoskeleton dysregulation, we also observed a highly significant enrichment of gene ontology (GO) terms concerning cell adhesion, by both integrins and cell junctions, and to neurogenesis (axonal guidance and synaptogenesis) and neurotransmission (GABAergic transmission).

Finally, to functionally correlate the differentially abundant detected proteins, pathway analyses were attempted by applying the STRING and MetaCore resources.

**Figure 5:**
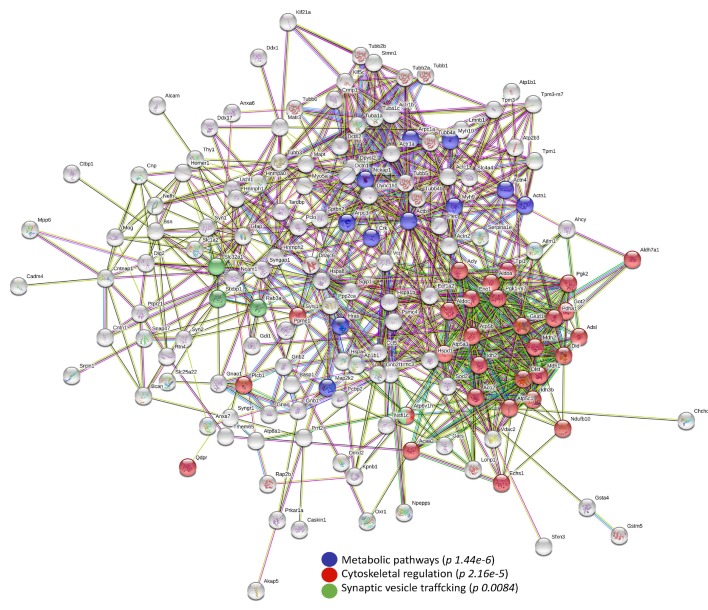
Protein–protein interaction (PPI) in murine NAGLU^−/−^ brain tissue proteome. The PPI network was explored by STRING (Search Tool for the Retrieval of Interacting Genes) software. The cluster analysis shows “Metabolic Pathways” (*p* 1.44 × 10^−6^), “Cytoskeletal Regulation” (*p* 2.16 × 10^−5^) and “Synaptic vesicle trafficking” (*p* 8.40 × 10^−3^) as significant pathways according to KEGG (Kyoto Encyclopedia of Genes and Genomes) database.

Noteworthy, the STRING protein–protein interaction (PPI) network showed the identified differences clustering in three main paths, that are all related to the above described enrichment analyses: cytoskeletal regulation, metabolism, and synaptic vesicle trafficking (Figure 5). These functional clusters highlight the fundamental role that defects in cytoskeletal organization, synaptic transmission, and energy balance act in MPS IIIB neuropathogenesis.

**Figure 6:**
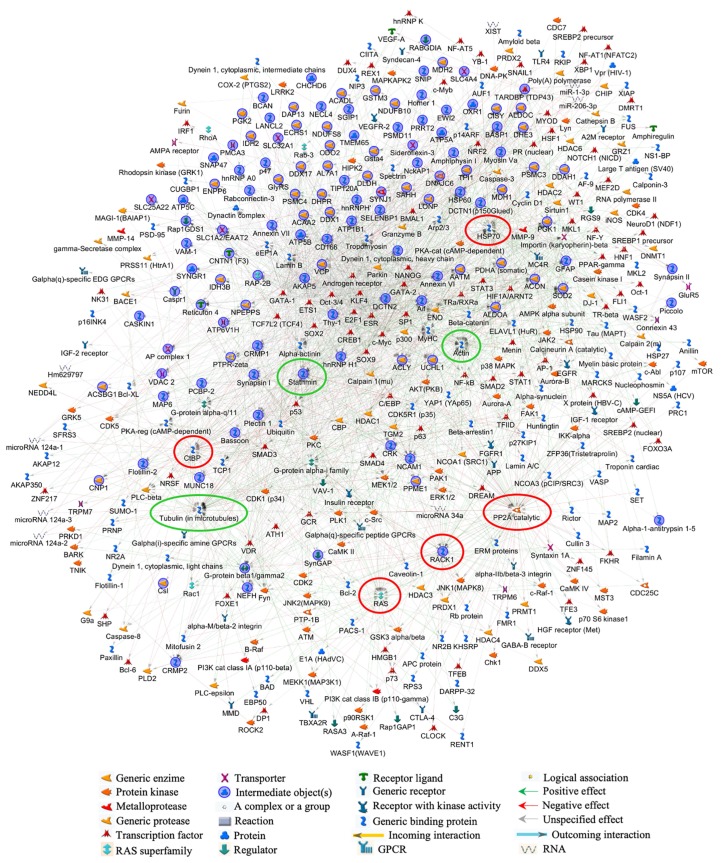
MetaCore protein network of murine NAGLU^−/−^ brain proteome. The protein–protein interactions characterizing the murine NAGLU^−/−^ brain proteome, were explored by MetaCore tools. The experimental proteins (blue circles) were processed according to known protein–protein interactions and other features established in the literature. The relationships existing between individual proteins and their directions were represented by arrows and lines. The following line colors designate the nature of the interaction: red = negative effect, green = positive effect, gray = unspecified effect.

The involvement of neuronal plasticity and signal transduction affections, along with an evident dysfunction in cytoskeleton, and even nucleoskeleton organization, with known degenerative consequences for the CNS, are definitively indicated by the MetaCore shortest path analysis as key processes in the neurological manifestations of MPS IIIB. According to the selected parameters, the built net resulted from the tight functional correlation existing among the processed EntrezGene-list corresponding proteins. Of relevance, about 80% of the processed differences entered into the net, thus proving the significance of the obtained data and the biological relevance of their deregulated abundance. Five main central hubs in the net (Figure 6, red circles) resulted: serine/threonine protein phosphatase 2A, catalytic subunit, alpha isoform (collapsed in PP2A catalytic in the MetaCore net; P63330; Fold_NSAF_ = 1.4), receptor for activated protein C kinase 1 (RACK1; P68040; Fold_NSAF_ = −1), C-terminal-binding protein 1 (collapsed in CtBP1 in the MetaCore net; O88712; Fold_NSAF_ = 0.6), GTPase HRas (collapsed in RAS in the MetaCore net; Q61411; Fold_NSAF_ = −2.2), HSC70 (collapsed in HSP70 in the MetaCore net—the protein difference heat shock cognate 71 kDa protein (hspa8; P63017; Fold_NSAF_ = 0.4) also functionally collapsed in this central hub). Despite that they were not designed among the above central hubs, tubulin (in microtubules), actin, and stathmin (Figure 6, green circles) act in key roles for the network by assuming central positions and interacting with several other proteins.

## 4. Discussion

**Figure 7:**
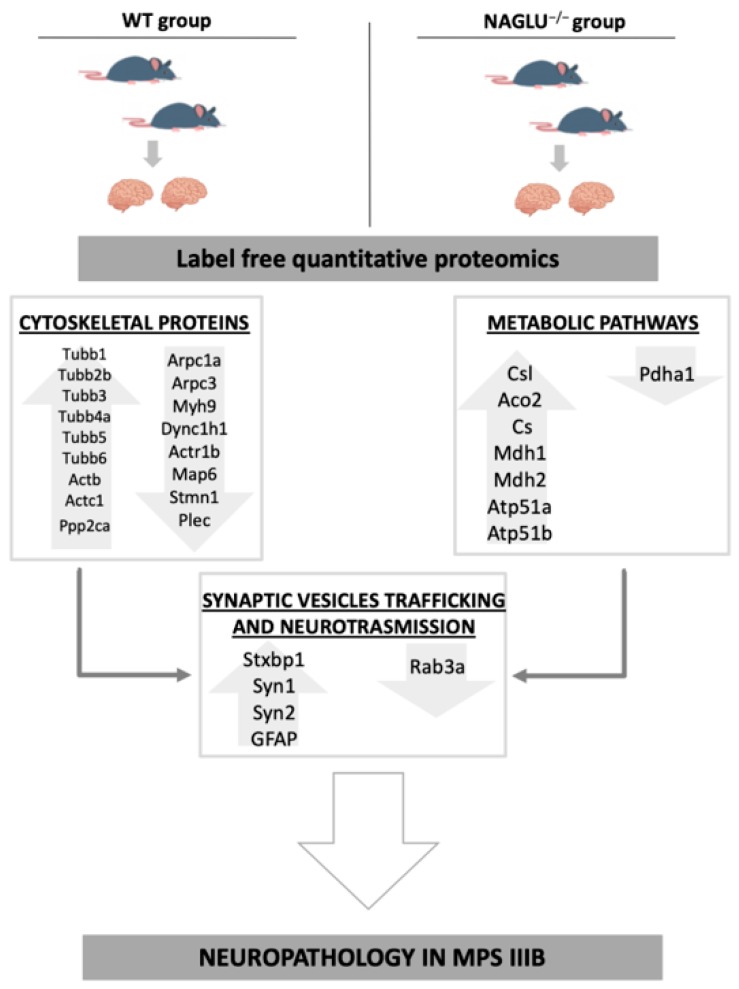
Representation of the main proteomic modifications induced by NAGLU deletion in murine brains. Dysregulation of cytoskeleton associated proteins and the energy metabolism-involved proteins may contribute to impair the lysosomal membrane trafficking pathway linked to the pathogenesis of neuropathy in MPS IIIB disease. Both altered pathways are relevant to preserve brain homeostasis and could be responsible for an impairment of synaptic vesicle formation and activity in the MPS IIIB mouse brain.

A label-free quantitative proteomic approach was employed to identify 204 proteins whose expression was found deregulated in NAGLU knockout murine brain tissues versus WT mice. Multiple bioinformatic analyses allowed us to classify these proteins into three major groups of biological processes: regulation of cytoskeleton organization, synaptic vesicle trafficking, and energy metabolism. Here we discuss the proteins that we identified as deregulated in NAGLU^−/−^ brains (Figure 7) within these biological processes and their involvement in the neuropathogenesis of MPS IIIB disease.

### 4.1. Deregulated Proteins Involved in Cytoskeleton Organization

The major group of dysregulated proteins in MPS IIIB mouse brain is made by cytoskeletal proteins. We found altered levels of a cluster of proteins involved in the cytoskeletal organization composed by Tubb (tubulin beta) 1, Tubb2b, Tubb3, Tubb4a, Tubb5, Tubb6, Actb (actin beta), Actc1 (actin alpha cardiac muscle 1), Arpc1a (actin-related protein 2/3 complex subunit 1a), Arpc3 (actin-related protein 2/3 complex subunit 3), and Myh9 (myosin-9). Furthermore, the cytoskeletal proteins dynein, Actr, Map6, Stmn1, plectin, and Ppp2cA were also found to be deregulated in MPS IIIB mouse brains.

In the developing brain, a large number of tubulin isoforms are expressed in neurons during neuronal migration and differentiation. They are globular proteins forming heterodimers that coassemble into microtubules, important cytoskeletal polymers that are involved in fundamental cellular processes such as cell division, motility, differentiation, intracellular cargo transport, and communications [44,45]. Distinct alpha- and beta-tubulin isoforms are required for the positioning, differentiation, and survival of neurons [46,47]. Mutations in genes encoding for alpha-tubulin (Tuba1a) and beta-tubulin (Tubb2a, Tubb3, Tubb4a) have been associated with a variety of brain malformations, including different types of cortical phenotypes [48]. As the deregulation of the tubulin isotype, Tubb4b was also found in the brain of MPS I mice [49]; the deregulation of tubulin isoforms Tubb1, Tubb2a, Tubb3, Tubb4a, Tubb5, and Tubb6 found in the MPS IIIB mouse brain strongly suggests that microtubule dysfunction may underlie the pathogenic mechanisms of neurological disorders in specific MPS subtypes.

Indeed, microtubule involvement in the MPS neurological component is supported by our results where we found altered expression levels of proteins such as Stathmin (Stmn1) and microtubule-associated protein 6 (Map6) detected in MPS IIIB mouse brain. Stmn1 has been shown to act as a key mediator in neuronal transduction pathways, and it is involved in the physiological regulation of microtubule destabilization. Moreover, it plays a critical role in the pathology of neurodegeneration such as Alzheimer’s disease (AD) [50]. Finally, Stmn1 regulates physiological development of Purkinje cell dendrites, controls the microtubule polymerization, and mediates the development of dendritic arbors in neuronal cells [51,52]. Map6 is deputed to stabilize neuronal microtubules and plays an important role in establishing axon–dendrite polarity [53]. Interestingly, we observed also the increased levels of Serine/threonine-protein phosphatase 2A catalytic subunit alpha isoform (Ppp2ca), described as the major phosphatase for microtubule-associated proteins (MAPs). Ppp2ca is the central factor in the NAGLU^−/−^ proteome, given that it establishes the greatest number of functional connections with the other proteins in the dataset hubs as shown in the net. Furthermore, the interaction of Map6 with the lysosomal protein TMEM 106B is crucial for controlling lysosomal trafficking by acting as a molecular brake for retrograde transport [54]. Indeed, lysosomes receive inputs from both endocytic and autophagic pathways, and release degraded products to Golgi apparatus through retrograde trafficking or to the extracellular space through exocytosis [55,56]. Thus, lysosomal misrouting may be responsible for neurodegeneration in MPS diseases. Consistently, in MPS IIIB mouse brains, we also found altered expression levels of the motor protein dynein complex which mediates lysosome movement towards the microtubule-minus ends (retrograde transport). The lysosome retrograde transport mediated by dynein requires the simultaneous binding of the protein to its adaptor dynactyn [57]. An impairment of dynein-mediated retrograde transport and clearance of autophagic vacuoles has been found in several neurodegenerative disorders [58].

Notably, in MPS IIIB mouse brains, we found also the deregulation of plectin, a protein that acts as the main linker of the intermediate filaments with microtubules and microfilaments. Plectin is a member of a structural family of proteins able to interlink different cytoskeletal elements. It might be involved not only in the cross-linking and stabilization of cytoskeletal intermediate filaments network, but also in the dynamic regulation of the cytoskeleton [59,60].

Overall, these findings suggest that the impairment of the lysosomal membrane trafficking pathway due to a deregulation of cytoskeleton-associated proteins may contribute to the pathogenesis of neuropathy in MPS IIIB disease.

### 4.2. Deregulated Proteins Involved in Metabolic Pathways

A group of proteins deregulated in murine NAGLU knockout brain tissue clustered as “TCA cycle” (Csl, Aco2, Pdha1, Cs, Mdh1), “Pyruvate metabolism” (Csl, Pdha1, Cs, Mdh2), and ATP Synthesis (Atp51a, Atp51b). These alterations strictly correlate with the deregulation of the lysosomal–autophagy pathway. Pharmacological inhibition of this pathway in cultured primary rat cortical neurons showed alterations in TCA cycle intermediates, particularly those downstream of citrate synthase and those linked to glutaminolysis [61]. Furthermore, autophagy impairment affects the quality and activity of mitochondria, including its electron transport chain function [61]. It has been shown that neurons of the mouse model of MPS IIIB accumulate the subunit c of mitochondrial ATP synthase (SCMAS) [62]. On the other hand, mitochondria engulf lysosomes by autophagy [63], a process that is critical for neuronal survival [64]. Suppression of autophagy in neural cells causes neurodegeneration in mice [64,65]. An aberrant lysosomal–autophagy pathway associated with neurodegeneration has been ascertained in various lysosomal storage diseases, including MPSs [31,66,67].

The mechanistic target of rapamycin complex 1 (mTORC1) plays a key role in maintaining cellular homeostasis by regulating metabolic processes [68]. Indeed, in nutrient-rich conditions, mTORC1 stimulates biosynthetic pathways (anabolic metabolism) and inhibits catabolic pathways. Acting in concert with the energy sensor AMP-activated protein kinase (AMPK) [58], mTORC1 drives anabolic or catabolic processes. Lysosomes, among their functions, serve also as platforms for both anabolic or catabolic signaling mediated by mTORC1 and AMPK [69]. These pathways are particularly relevant for maintaining brain homeostasis, and increasing evidence suggests that metabolic alterations strongly influence the initiation and progression of neurodegenerative disorders [70,71].

Our results, that identify dysregulated proteins involved in energy metabolism in MPS IIIB mouse brain, strongly suggest the involvement of metabolic pathway alterations in the development of neurological phenotypes in the MPS IIIB disease.

### 4.3. Deregulated Proteins Involved in Synaptic Vesicle Trafficking and Neurotransmission

Energy metabolism is relevant for neuronal plasticity, axonal transport, synaptic vesicle trafficking and docking, and thereby neurotransmitter release [72]. In our study, dysregulation of the expression levels of proteins involved in “Synaptic vesicle trafficking” and in “neurotransmitter release” pathways, including the syntaxin-binding protein 1 (Stxbp1), synapsin 1 and 2 (Syn1, Syn2), and Rab3a, was observed in the brain of MPS IIIB mice.

The brain membrane transport protein Stxbp1, also known as Munc18-1, is a key component of synaptic vesicle-fusion machinery, thus playing an important role in neurotransmitter secretion [73]. Heterozygous de novo mutations in the neuronal protein Munc18-1 are associated with epilepsies, movement disorders, intellectual disability, and neurodegeneration [74].

Synapsins (Syns) are the most abundant protein family present on synaptic vesicles and are phosphorylated at multiple sites by various protein kinases [75]. When dephosphorylated Syns are associated with the synaptic vesicle membrane, while when phosphorylated they dissociate from the synaptic vesicles thus stimulating the exocytosis [76,77]. Three isoforms of Syns, namely Syn 1, Syn 2, and Syn 3, are highly expressed in nerve cells, but Syn 1 and Syn 2 are the major isoforms in neurons. They are involved in the elongation of axons, formation of presynaptic terminals, regulation of the vesicle reserve pool at presynaptic terminals, synaptogenesis, and synaptic vesicle docking [78,79]. These proteins display a highly conserved ATP binding site in the central C-domain, and this binding modulates synaptic vesicle clustering and plasticity of inhibitory synapses [80]. The Syn-dependent cluster of synaptic vesicles plays a key role in sustaining the release of neurotransmitter in response to high levels of neuronal activity [75]. Indeed, Syn1 null mice exhibit altered synaptic vesicle organization at presynaptic terminals coupled to a reduced neurotransmitter release, and delayed recovery of synaptic transmission after neurotransmitter depletion [76]. Abnormal expression/activity of Syns has been associated with several neurological disorders including epilepsy, schizophrenia, Huntington’s disease, Alzheimer’s disease, multiple sclerosis (MS), and autism [78,79].

Finally, in MPS IIIB mouse brain, we found altered expression levels of Rab3a, a GTPase protein which localizes to the synaptosomes and secretory granules. This protein is a critical player in the regulation of secretion and neurotransmitter release [81]. In general, Rab GTPases are involved in the control of vesicular traffic by recruiting effector proteins that bind exclusively to the GTP-bound, active form of the GTPase [82]. Several evidences demonstrate that Rab3a interacts with the cortical actin cytoskeleton via its effector, the nonmuscle myosin heavy chain IIA (NMHC IIA), an actin-dependent motor adaptor responsible for the positioning of the lysosomes at the periphery of the cell [83].

Previous studies in the mouse model of the Gaucher lysosomal storage disease have shown a deregulation of dopamine neurotransmission indicative of synaptic dysfunction [84]. Altered dopamine transport system imaging resulted to be pathologic in Niemann-Pick type C-case reports [85,86]. These findings together with our observations of a deregulation of proteins involved in synaptic vesicle formation and activity in the MPS IIIB mouse brain strongly suggest the involvement of synaptic dysfunction and impaired neurotransmission in the pathogenesis of the neurological disorders associated with some MPS subtypes and other lysosomal storage diseases as well.

### 4.4. Other Proteins

Altered expression levels of Glial fibrillary acidic protein (GFAP) were found in the MPS IIIB mouse brain. This finding is consistent with previous studies that highlighted a deregulation of GFAP in several lysosomal storage diseases, including MPS IIIB [18,87,88,89,90,91,92,93,94,95]. The protein GFAP is a molecular marker of astrocytes that are fundamental for the neuronal microenvironment in order to control the metabolism of glucose, neurotransmitters re-uptake, and the formation and maturation of the synapses. Moreover, astrocytes play a crucial role in inflammation and neurodegeneration in the brain. Indeed, they are the source and the target of inflammatory cytokines together with the microglia and oligodendrocytes. Elevated levels of proinflammatory cytokines such as IL-1, TNF-α, MCP-1, and MIP-1α have been found in the CNS of MPS murine models [20,90,96,97] as well as in the mouse models of Gaucher disease and Krabbe [98]. Although most of these cytokines might be released by the activated microglia, activated astrocytes and neurons are also capable to diffuse proinflammatory cytokine signaling. Significant increased levels of MCP-1, MIP-1α, and IL-1α were observed in MPS I and MPS III mouse brains [90,99,100]. Upregulation of TNF-α and TNFR1 gene expression was detected in MPS IIIB mouse brain [96,101]. Serum and synovial fluid levels of TNF-α were found increased in MPS VII mice and dogs, respectively [93,94]. These findings suggest the activation of signaling from the TLRs and IL-1 receptors, which together would contribute to TNF-α production. IL-1β levels were found upregulated in both MPS IIIA and MPS IIIB brains [20,96], in MPS VII dogs synovial fluid, and MPS VI rat fibroblast-like synoviocytes [102,103,104]. Innate immunity appears to have a dominating role in MPSs by controlling lipid metabolism, glycosaminoglycan degradation, autophagy, and regulation of the cytokines release by the inflammasome [95]. Moreover, the relationship between inflammation and autophagy has been recently linked to the release of the specific IL-1β by the inflammasome [104]. In our study, we also found proteins involved in inflammation dysregulated in the MPS IIIB mouse brains.

## 5. Conclusions

In this work we analyze for the first time the differences in the proteome profiles between brains from MPS IIIB vs. WT mice and we highlight that alterations in metabolic pathways, organelle homeostasis, and cytoskeletal system may play a fundamental role in the neuropathology of MPS IIIB. Deregulation of cytoskeleton and energy metabolism-associated proteins may contribute to impair the lysosomal membrane trafficking pathway linked to the pathogenesis of neuropathy in MPS IIIB disease. Both altered pathways are relevant to preserve brain homeostasis and could be responsible for the impairment of synaptic vesicle formation and activity in the MPS IIIB mouse brain. We believe that an in depth shotgun proteomic analysis would be required to validate this first study on protein abundance quantifications in the MPS IIIB mouse model. In addition, future studies will be necessary to evaluate the involvement of specific pathways which could be the molecular basis of the neuropathology seen in MPS IIIB patients and animal models. Furthermore, it would be of great interest to investigate the proteomic profiles of brains from other lysosomal diseases and compare them together in order to find common hallmarks of these neurological diseases.

## Figures and Tables

**Table 1:** Dysregulated proteins in NAGLU^−/−^ mouse brains compared to WT.

Swiss-Prot Code	Gene Name	Protein Description	Fold NSAF	*p* Value	Subcellular Localization
Q545B6	Stmn1	Stathmin	−12.6	0.00002	CK
Q9QYX7	Pclo	Protein piccolo	−8.4	0.00101	CK, GA
Q6P9K8	Caskin1	Caskin-1	−3.8	0.00262	CK
A8DUK2	Hbbt1	Beta-globin	−3.4	0.00008	C
Q4VAE3	Tmem65	Transmembrane protein 65	−3.3	0.00374	M, PM
Q8CHF1	mKIAA0531	Kinesin-like protein (Fragment)	−3.2	0.00232	CK
P17563	Selenbp1	Selenium-binding protein 1	−3.2	0.00046	CK, N
Q4KMM3	Oxr1	Oxidation resistance protein 1	−3	0.00036	M, N
A2AS98	Nckap1	Nck-associated protein 1	−2.9	0.00209	CK, PM
C7G3P1	Ppfia3	MKIAA0654 protein (Fragment)	−2.6	0.00328	CK
Q8BVQ5	Ppme1	Protein phosphatase methylesterase 1	−2.5	0.00345	N
Q3UVN5	Nsfl1c	Putative uncharacterized protein	−2.5	0.00002	CK, GA, N
Q9R0Q6	Arpc1a	Actin-related protein 2/3 complex subunit 1A	−2.4	0.00036	CK, N, PM
Q3TF14	Ahcy	Adenosylhomocysteinase	−2.4	0.00329	CK, N
Q9CX86	Hnrnpa0	Heterogeneous nuclear ribonucleoprotein A0	−2.4	0.00165	N
Q9Z1B3	Plcb1	1-phosphatidylinositol 4,5-bisphosphate phosphodiesterase beta-1	−2.4	0.00671	CK, N, PM
O54991	Cntnap1	Contactin-associated protein 1	−2.3	0.00626	PM
B0V2P5	Dmxl2	DmX-like protein 2	−2.3	0.01109	PM
E9Q8N8	Slc4a4	Anion exchange protein	−2.3	0.0011	PM
B1AQX9	Srcin1	SRC kinase-signaling inhibitor 1	−2.3	0.0004	CK
P24472	Gsta4	Glutathione S-transferase A4	−2.3	0.00125	M
Q3UK83	Hnrnpa1	Putative uncharacterized protein	−2.2	0.00047	ERS, N
D3Z4J3	Myo5a	Unconventional myosin-Va	−2.2	0.00636	CK, C, ER, En, GA, Ly, Pe
Q61411	Hras	GTPase HRas	−2.2	0.00583	C, GA, N, PM
Q9Z0X1	Aifm1	Apoptosis-inducing factor 1, mitochondrial	−2.1	0.00398	CK, M, N
B2L107	Vsnl1	Visinin-like protein 1	−2.1	0.00018	C
Q9DBF1	Aldh7a	Alpha-aminoadipic semialdehyde dehydrogenase	−2.1	0.02264	C, M, N
Q3TJF2	Ola1	Obg-like ATPase 1	−2.1	0.00001	CK, C, N
Q8VDD5	Myh9	Myosin-9	−2	0.01358	C, CK, N, PM
Q9JJK2	Lancl2	LanC-like protein 2	−2	0.00232	C, CK, N, PM
E9Q2L2	Dlg2	Disks large homolog 2	−2	0.00218	PM
Q91VR5	Ddx1	ATP-dependent RNA helicase DDX1	−2	0.00216	C, M, N
Q9JHU4	Dync1h1	Cytoplasmic dynein 1 heavy chain 1	−2	0.00407	CK, N
Q99PU5	Acsbg1	Long-chain-fatty-acid--CoA ligase ACSBG1	−1.9	0.01942	ER, PM
Q64010	Crk	Adapter molecule crk	−1.9	0.0031	CK, PM
P14733	Lmnb1	Lamin-B1	−1.9	0.02293	CK, N
P54775	Psmc4	26S protease regulatory subunit 6B	−1.9	0.03099	CK, N
A0A1S6GWI0	Ndufa8	NADH dehydrogenase (Ubiquinone) 1 alpha subcomplex, 8	−1.8	0.00156	M
Q3U741	Ddx17	DEAD (Asp-Glu-Ala-Asp) box polypeptide 17, isoform CRA_a	−1.8	0.00085	C, N
Q3TIQ2	Rpl12	Putative uncharacterized protein	−1.8	0.01139	C, N
P70168	Kpnb1	Importin subunit beta-1	−1.7	0.00011	C, M, N
Q80TZ3	Dnajc6	Putative tyrosine-protein phosphatase auxilin	−1.7	0.00164	C
Q5DTG0	Atp8a1	Phospholipid-transporting ATPase (Fragment)	−1.7	0.00428	ER, GA, PM
Q9QXS1	Plec	Plectin	−1.7	0.00377	C, CK, PM
Q921F2	Tardbp	TAR DNA-binding protein 43	−1.7	0.00786	N
B2RX08	Sptb	Spectrin beta chain	−1.7	0.00319	C, CK, PM
Q3TKG4	Psmc3	Putative uncharacterized protein (Fragment)	−1.6	0.00396	N
Q3UH59	Myh10	Myosin-10	−1.6	0.02527	C, CK, PM
Q9JM76	Arpc3	Actin-related protein 2/3 complex subunit 3	−1.6	0.01437	C, N
A0A1S6GWJ8		Uncharacterized protein	–1,5	0.02713	
Q3UN60	Mpp6	Membrane protein, palmitoylated 6 (MAGUK p55 subfamily member 6), isoform CRA_b	−1.5	0.00085	PM
Q91XV3	Basp1	Brain acid soluble protein 1	−1.5	0.00001	N, PM
Q07076	Anxa7	Annexin A7	−1.5	0.01183	C, ER, ERS, N, PM
Q3UY05	Ndufs8	Putative uncharacterized protein	−1.4	0.00008	M
A0A0R4J0Q5	Lmnb2	Lamin-B2	−1.4	0.03275	CK, N
Q4FJX9	Sod2	Superoxide dismutase	−1.4	0.01379	M
Q91VN4	Chchd6	MICOS complex subunit Mic25	−1.4	0.01573	C, M
O88737	Bsn	Protein bassoon	−1.3	0.00915	C, GA
Q3UWW9	Psmd11	Putative uncharacterized protein	−1.3	0.01386	C, N
O08788	Dctn1	Dynactin subunit 1	−1.3	0.01243	CK, C, ERS, N
A0A0R4J275	Ndufa12	NADH dehydrogenase [ubiquinone] 1 alpha subcomplex subunit 12	−1.3	0.0197	C, M
Q63932	Map2k2	Dual specificity mitogen-activated protein kinase kinase 2	−1.3	0.01951	CK, C, ER, En, GA, M, N, PM
Q61361	Bcan	Brevican core protein	−1.3	0.01264	ERS
Q3V117	Acly	ATP-citrate synthase	−1.3	0.0078	C, M, N, PM
Q8R570	Snap47	Synaptosomal-associated protein 47	−1.3	0.00248	C, PM
Q9DCS9	Ndufb10	NADH dehydrogenase [ubiquinone] 1 beta subcomplex subunit 10	−1.2	0.01688	M
P14824	Anxa6	Annexin A6	−1.2	0.01378	C, En, ERS, Ly, M, N, PM
P54071	Idh2	Isocitrate dehydrogenase [NADP], mitochondrial	−1.2	0.00039	C, M, Pe
Q7TSJ2	Map6	Microtubule-associated protein 6	−1.2	0.00159	CK, GA
Q8BGN3	Enpp6	Ectonucleotide pyrophosphatase/phosphodiesterase family member 6	−1.2	0.03099	ERS, PM
Q9CZD3	Gars	Glycine--tRNA ligase	−1.2	0.01474	C, ERS, M
A0A0R4J083	Acadl	Long-chain-specific acyl-CoA dehydrogenase, mitochondrial	−1.2	0.03611	M
Q8CGK3	Lonp1	Lon protease homolog, mitochondrial	−1.1	0.01936	C, M, N
E9Q7Q3	Tpm3	Tropomyosin alpha-3 chain	−1.1	0.00355	CK, C
Q8BWT1	Acaa2	3-ketoacyl-CoA thiolase, mitochondrial	−1.1	0.04478	M
P61226	Rap2b	Ras-related protein Rap-2b	−1.1	0.03025	C, En, ERS, PM
Q8R5C5	Actr1b	Beta-centractin	−1.1	0.00172	CK
E9Q0J5	Kif21a	Kinesin-like protein KIF21A	−1.1	0.04345	CK, C, PM
B9EKR1	Ptprz1	Receptor-type tyrosine-protein phosphatase zeta	−1.1	0.01214	ERS, PM
Q6ZQ38	Cand1	Cullin-associated NEDD8-dissociated protein 1	−1	0.00177	C, GA, N
Q9JI91	Actn2	Alpha-actinin-2	−1	0.00286	CK, PM
P68040	Rack1	Receptor of activated protein C kinase 1	−1	0.03823	CK, M, N, PM
E9PUL5	Prrt2	Proline-rich transmembrane protein 2	−1	0.0411	PM
F6SEU4	Syngap1	Ras/Rap GTPase-activating protein SynGAP	−1	0.03268	PM
Q8CHG1	Dclk1	MKIAA0369 protein (Fragment)	−1	0.02306	PM
Q61490	Alcam	CD166 antigen	−1	0.03414	PM
D3Z656	Synj1	Synaptojanin-1	−1	0.02126	PM
H3BIV5	Akap5	A-kinase anchor protein 5	−1	0.01709	CK, PM
Q8VD37	Sgip1	SH3-containing GRB2-like protein 3-interacting protein 1	−1	0.04017	CK, PM
W6PPR4	Ank3	480-kDa ankyrinG	−1	0.00579	CK, C, PM
Q9CWS0	Ddah1	N(G),N(G)-dimethylarginine dimethylaminohydrolase 1	−0.9	0.04649	M
Q11011	Npepps	Puromycin-sensitive aminopeptidase	−0.9	0.00505	C, N
Q3TXE5	Canx	Putative uncharacterized protein	−0.9	0.00488	ER, PM
Q3UAG2	Pgd	6-phosphogluconate dehydrogenase, decarboxylating	−0.9	0.02403	C
Q99JX6	Anxa6	Annexin	−0.9	0.04313	C, En, ERS, Ly, M, N, PM
A0A0G2JEG8	Amph	Amphiphysin	−0.9	0.00912	CK, PM
Q99P72	Rtn4	Reticulon-4	−0.9	0.01061	ER, N, PM
Q49S98	Slc32a1	Putative uncharacterized protein	−0.9	0.04098	PM
B2RQQ5	Map1b	Microtubule-associated protein 1B	−0.9	0.03443	CK, C, PM
D3Z2H9	Tpm3	Uncharacterized protein	−0.9	0.02982	CK, C
Q6ZQ61	Matr3	MCG121979, isoform CRA_c (Fragment)	−0.8	0.01811	N
Q3TE45	Sdhb	Succinate dehydrogenase [ubiquinone] iron-sulfur subunit, mitochondrial	−0.8	0.01667	M, N, PM
Q0VF55	Atp2b3	Calcium-transporting ATPase	−0.8	0.03416	PM
Q3UEG9	Flot2	Putative uncharacterized protein	−0.8	0.02632	CK, En, PM
Q91V61	Sfxn3	Sideroflexin-3	−0.8	0.00937	M
P61164	Actr1a	Alpha-centractin	−0.8	0.03531	CK
Q571M2	Hspa4	MKIAA4025 protein (Fragment)	−0.7	0.00487	C, ERS, N
Q9Z2Y3	Homer1	Homer protein homolog 1	−0.7	0.01584	C, PM
E9Q455	Tpm1	Tropomyosin alpha-1 chain	−0.7	0.04225	C
A2A5Y6	Mapt	Microtubule-associated protein	−0.7	0.04838	CK, C, N, PM
A0A1S6GWH1		Uncharacterized protein	–0,7	0.02404	
P35486	Pdha1	Pyruvate dehydrogenase E1 component subunit alpha, somatic form, mitochondrial	−0.6	0.00552	M, N
Q8CC13	Ap1b1	AP complex subunit beta	−0.6	0.03216	C, GA
E9Q2W9	Actn4	Alpha-actinin-4 (Fragment)	−0.6	0.00847	CK, C, N
Q8BVE3	Atp6v1h	V-type proton ATPase subunit H	−0.6	0.02175	C, Ly
A0A0A6YY91	Ncam1	Neural cell adhesion molecule 1 (Fragment)	−0.6	0.0182	CK, PM
Q3TYK4	Prkar1a	Putative uncharacterized protein	−0.6	0.01177	CK, C, PM
P12960	Cntn1	Contactin-1	−0.6	0.02931	PM
Q3TPZ5	Dctn2	Dynactin 2	−0.6	0.00054	CK, C
A0A1L1SV25	Actn4	Alpha-actinin-4	−0.6	0.0286	CK, C, N
P19246	Nefh	Neurofilament heavy polypeptide	−0.5	0.04106	CK, M, N, PM
A0A0R4J117	Igsf8	Immunoglobulin superfamily member 8	−0.5	0.04908	PM
Q68FG2	Sptbn2	Spectrin beta chain	−0.5	0.04372	CK, C, ER, En, GA
Q80YU5	Mog	Myelin oligodendrocyte glycoprotein (Fragment)	−0.5	0.02477	C, ER, M, PM
E9QB01	Ncam1	Neural cell adhesion molecule 1	−0.5	0.03648	C, PM
Q01853	Vcp	Transitional endoplasmic reticulum ATPase	−0.5	0.03249	C, ER, N
P09041	Pgk2	Phosphoglycerate kinase 2	−0.4	0.03696	C
Q7TPR4	Actn1	Alpha-actinin-1	−0.4	0.01557	CK, N, PM
P63011	Rab3a	Ras-related protein Rab-3A	−0.4	0.0162	C, En, Ly, PM
P09411	Pgk1	Phosphoglycerate kinase 1	−0.3	0.01617	C, ERS, PM
O08599	Stxbp1	Syntaxin-binding protein 1	0.1	0.03134	CK, C, M, PM
A0A0A0MQA5	Tuba4a	Tubulin alpha chain (Fragment)	0.2	0.01472	CK
P68369	Tuba1a	Tubulin alpha-1A chain	0.2	0.02299	CK, En
Q52L87	Tuba1c	Tubulin alpha chain	0.3	0.0146	CK, N
P50396	Gdi1	Rab GDP dissociation inhibitor alpha	0.3	0.03464	GA
P60710	Actb	Actin, cytoplasmic 1	0.3	0.02793	CK, C, N, PM
Q9CZU6	Cs	Citrate synthase, mitochondrial	0.3	0.00075	M
P05202	Got2	Aspartate aminotransferase, mitochondrial	0.3	0.03387	M, PM
P63038	Hspd1	60 kDa heat shock protein, mitochondrial	0.3	0.02215	CK, ER, En, ERS, GA, M, PM
E9Q912	Rap1gds1	RAP1, GTP-GDP dissociation stimulator 1	0.3	0.00467	CK, ER, M
B2CSK2		Heat shock protein 1-like protein	0.3	0.00172	
O88935	Syn1	Synapsin-1	0.3	0.03624	CK, C, GA, N
P68033	Actc1	Actin, alpha cardiac muscle 1	0.3	0.00986	CK
P08249	Mdh2	Malate dehydrogenase, mitochondrial	0.3	0.00731	M
Q03265	Atp5a1	ATP synthase subunit alpha, mitochondrial	0.3	0.00169	M, N, PM
Q99KI0	Aco2	Aconitate hydratase, mitochondrial	0.3	0.00087	C, M
P17879	Hspa1b	Heat shock 70 kDa protein 1B	0.4	0.00362	CK, C, M, N, PM
O08553	Dpysl2	Dihydropyrimidinase-related protein 2	0.4	0.00004	CK, C, M, PM
Q5FW97	EG433182	Enolase 1, alpha non-neuron	0.4	0.00346	C
Q64332	Syn2	Synapsin-2	0.4	0.01708	PM
Q3TQ70	Gnb1	Beta1 subnuit of GTP-binding protein	0.4	0.01897	PM
P62631	Eef1a2	Elongation factor 1-alpha 2	0.4	0.00011	N
P80316	Cct5	T-complex protein 1 subunit epsilon	0.4	0.04942	CK, C
Q8CE19	Syn2	Putative uncharacterized protein	0.4	0.02198	PM
Q3UA81	Eef1a1	Elongation factor 1-alpha	0.4	0.00003	CK, C, M, PM
Q8C2Q7	Hnrnph1	Heterogeneous nuclear ribonucleoprotein H	0.4	0.01261	C, N
Q9D6F9	Tubb4a	Tubulin beta-4A chain	0.4	0.00031	CK
P17751	Tpi1	Triosephosphate isomerase	0.4	0.00008	C
P48774	Gstm5	Glutathione S-transferase Mu 5	0.4	0.01045	C, ERS
P18872	Gnao1	Guanine nucleotide-binding protein G(o) subunit alpha	0.4	0.00334	PM
Q3UYK6	Slc1a2	Amino acid transporter	0.4	0.03711	PM
Q8BVI4	Qdpr	Dihydropteridine reductase	0.4	0.02048	C, M
Q9D2G2	Dlst	Dihydrolipoyllysine-residue succinyltransferase component of 2-oxoglutarate dehydrogenase complex, mitochondrial	0.4	0.00639	M, N, PM
Q3UD06	Atp5c1	ATP synthase subunit gamma	0.4	0.00728	M
P68372	Tubb4b	Tubulin beta-4B chain	0.4	0.00008	CK
P99024	Tubb5	Tubulin beta-5 chain	0.4	0.00005	CK, C, M
P56480	Atp5b	ATP synthase subunit beta, mitochondrial	0.4	0.00166	M, PM
E9QKR0	Gnb2	Guanine nucleotide-binding protein G(I)/G(S)/G(T) subunit beta-2	0.4	0.0162	PM
B2RSN3	Tubb2b	Tubulin beta chain	0.4	0.00013	CK
Q7TMM9	Tubb2a	Tubulin beta-2A chain	0.4	0.0001	CK
P63017	Hspa8	Heat shock cognate 71 kDa protein	0.4	0.0001	CK, C, En, ERS, Ly, N, PM
Q3TIC8	Uqcrc1	Putative uncharacterized protein	0.4	0.00014	C, M
P26443	Glud1	Glutamate dehydrogenase 1, mitochondrial	0.4	0.00008	M
Q3TYV5	Cnp	2′,3′-cyclic-nucleotide 3′-phosphodiesterase	0.5	0.00001	ERS
B7U582		Heat shock protein 70-2	0.5	0.00001	
Q8BH95	Echs1	Enoyl-CoA hydratase, mitochondrial	0.5	0.03689	M
Q6P1J1	Crmp1	Crmp1 protein	0.5	0.00045	CK, C, N
B2CY77	Rpsa	Laminin receptor (Fragment)	0.5	0.01872	C, ERS, N, PM
Q922F4	Tubb6	Tubulin beta-6 chain	0.5	0.00002	CK
Q9ERD7	Tubb3	Tubulin beta-3 chain	0.5	0.00003	CK
Q60930	Vdac2	Voltage-dependent anion-selective channel protein 2	0.5	0.00247	M
P14152	Mdh1	Malate dehydrogenase, cytoplasmic	0.5	0.00092	C, M
A6ZI44	Aldoa	Fructose-bisphosphate aldolase	0.5	0.00156	CK, C, ERS, M, N, PM
Q80X68	Csl	Citrate synthase	0.5	0.00001	N
A2AQ07	Tubb1	Tubulin beta-1 chain	0.5	0.00009	CK
P01831	Thy1	Thy-1 membrane glycoprotein	0.6	0.00272	C, ER, PM
A0A1S6GWG6		Uncharacterized protein	0,6	0.00144	
Q3UBZ3	Capza2	Putative uncharacterized protein	0.6	0.03767	CK
Q9D6M3	Slc25a22	Mitochondrial glutamate carrier 1	0.6	0.02851	M
O08749	Dld	Dihydrolipoyl dehydrogenase, mitochondrial	0.6	0.00293	M, N
Q91VA7	Idh3b	Isocitrate dehydrogenase [NAD] subunit, mitochondrial	0.6	0.0105	M
O88712	Ctbp1	C-terminal-binding protein 1	0.6	0.00177	N
Q8C3L6	Atp6v1b1	Putative uncharacterized protein	0.6	0.04137	PM
P70333	Hnrnph2	Heterogeneous nuclear ribonucleoprotein H2	0.6	0.00158	N
P14094	Atp1b1	Sodium/potassium-transporting ATPase subunit beta-1	0.7	0.00001	PM
O55100	Syngr1	Synaptogyrin-1	0.7	0.03643	PM
Q9R0P9	Uchl1	Ubiquitin carboxyl-terminal hydrolase isozyme L1	0.7	0.00142	C, ER, PM
P05063	Aldoc	Fructose-bisphosphate aldolase C	0.7	0.00002	C, M
Q61990	Pcbp2	Poly(rC)-binding protein 2	0.8	0.03331	C, N
Q8R464	Cadm4	Cell adhesion molecule 4	0.8	0.01837	PM
P21279	Gnaq	Guanine nucleotide-binding protein G(q) subunit alpha	0.9	0.04159	C, N, PM
Q00898	Serpina1	Alpha-1-antitrypsin 1-5	1.2	0.04259	ER, ERS, GA
P63330	Ppp2ca	Serine/threonine-protein phosphatase 2A catalytic subunit alpha isoform	1.4	0.00163	CK, C, N, PM
P03995	Gfap	Glial fibrillary acidic protein	1.4	0.00001	CK, Ly

CK, Cytoskeleton; C, Cytosol; ER, Endoplasmic reticulum; En, Endosome; ERS, Extracellular region or secreted; GA, Golgi apparatus; Ly, Lysosome; M, Mitochondrion; N, Nucleus; Pe, Peroxisome PM, Plasma Membrane.

**Table 2:** REACTOME pathway classification of murine NAGLU^−/−^ brain tissue proteome.

REACTOME Pathways	*Mus musculus* REFLIST	Client Input	Client Input (Raw *p*-Value)
Microtubule-dependent trafficking of connexons from Golgi to the plasma membrane (R-MMU-190840)	14	7	1.45 × 10^−11^
Citric acid cycle (TCA cycle) (R-MMU-71403)	22	8	3.68 × 10^−12^
RHO GTPases activate IQGAPs (R-MMU-5626467)	25	8	8.61 × 10^−12^
Carboxyterminal post-translational modifications of tubulin (R-MMU-8955332)	25	7	4.00 × 10^−10^
Recycling pathway of L1 (R-MMU-437239)	34	9	1.74 × 10^−12^
Lysine catabolism (R-MMU-71064)	12	3	7.16 × 10^−05^
HSP90 chaperone cycle for steroid hormone receptors (SHR) (R-MMU-3371497)	51	12	1.00 × 10^−15^
COPI-independent Golgi-to-ER retrograde traffic (R-MMU-6811436)	47	10	6.42 × 10^−13^
Gluconeogenesis (R-MMU-70263)	35	7	3.07 × 10^−09^
Serotonin Neurotransmitter Release Cycle (R-MMU-181429)	17	3	1.76 × 10^−04^
Pyruvate metabolism and Citric Acid (TCA) cycle (R-MMU-71406)	51	9	4.21 × 10^−11^
GABA synthesis, release, reuptake and degradation (R-MMU-888590)	19	3	2.35 × 10^−04^
Dopamine Neurotransmitter Release Cycle (R-MMU-212676)	22	3	3.47 × 10^−04^
Glyoxylate metabolism and glycine degradation (R-MMU-389661)	30	4	3.64 × 10^−05^
Kinesins (R-MMU-983189)	54	7	4.54 × 10^−08^
Intraflagellar transport (R-MMU-5620924)	54	7	4.54 × 10^−08^
Recruitment of NuMA to mitotic centrosomes (R-MMU-380320)	86	11	6.28 × 10^−12^
RAF activation (R-MMU-5673000)	25	3	4.89 × 10^−04^
COPI-mediated anterograde transport (R-MMU-6807878)	98	11	2.29 × 10^−11^
The role of GTSE1 in G2/M progression after G2 checkpoint (R-MMU-8852276)	72	8	1.45 × 10^−08^
Loss of Nlp from mitotic centrosomes (R-MMU-380259)	67	7	1.77 × 10^−07^
AURKA Activation by TPX2 (R-MMU-8854518)	70	7	2.33 × 10^−07^
MHC class II antigen presentation (R-MMU-2132295)	120	12	9.50 × 10^−12^
Recruitment of mitotic centrosome proteins and complexes (R-MMU-380270)	76	7	3.91 × 10^−07^
Regulation of PLK1 Activity at G2/M Transition (R-MMU-2565942)	85	7	7.92 × 10^−07^
Hedgehog “on” state (R-MMU-5632684)	103	8	1.91 × 10^−07^
ER to Golgi Anterograde Transport (R-MMU-199977)	155	12	1.52 × 10^−10^
COPI-dependent Golgi-to-ER retrograde traffic (R-MMU-6811434)	92	7	1.30 × 10^−06^
Anchoring of the basal body to the plasma membrane (R-MMU-5620912)	93	7	1.40 × 10^−06^
RHO GTPases Activate Formins (R-MMU-5663220)	133	10	7.13 × 10^−09^
Resolution of Sister Chromatid Cohesion (R-MMU-2500257)	121	9	4.53 × 10^−08^
Hedgehog “off” state (R-MMU-5610787)	109	8	2.87 × 10^−07^
Glycolysis (R-MMU-70171)	63	4	5.22 × 10^−04^
Separation of Sister Chromatids (R-MMU-2467813)	185	10	1.36 × 10^−07^
Mitotic Anaphase (R-MMU-68882)	188	10	1.57 × 10^−07^
Neutrophil degranulation (R-MMU-6798695)	553	14	3.17 × 10^−06^
Microtubule-dependent trafficking of connexons from Golgi to the plasma membrane (R-MMU-190840)	14	7	1.45 × 10^−11^
Citric acid cycle (TCA cycle) (R-MMU-71403)	22	8	3.68 × 10^−12^
RHO GTPases activate IQGAPs (R-MMU-5626467)	25	8	8.61 × 10^−12^
Carboxyterminal post-translational modifications of tubulin (R-MMU-8955332)	25	7	4.00 × 10^−10^
Recycling pathway of L1 (R-MMU-437239)	34	9	1.74 × 10^−12^
Lysine catabolism (R-MMU-71064)	12	3	7.16 × 10^−05^
Fatty Acids bound to GPR40 (FFAR1) regulate insulin secretion (R-MMU-434316)	8	2	1.33 × 10^−03^
HSP90 chaperone cycle for steroid hormone receptors (SHR) (R-MMU-3371497)	51	12	1.00 × 10^−15^
COPI-independent Golgi-to-ER retrograde traffic (R-MMU-6811436)	47	10	6.42 × 10^−13^
Gluconeogenesis (R-MMU-70263)	35	7	3.07 × 10^−09^
Serotonin Neurotransmitter Release Cycle (R-MMU-181429)	17	3	1.76 × 10^−04^
Pyruvate metabolism and Citric Acid (TCA) cycle (R-MMU-71406)	51	9	4.21 × 10^−11^
GABA synthesis, release, reuptake and degradation (R-MMU-888590)	19	3	2.35 × 10^−04^
Dopamine Neurotransmitter Release Cycle (R-MMU-212676)	22	3	3.47 × 10^−04^
Glyoxylate metabolism and glycine degradation (R-MMU-389661)	30	4	3.64 × 10^−05^
Kinesins (R-MMU-983189)	54	7	4.54 × 10^−08^
Intraflagellar transport (R-MMU-5620924)	54	7	4.54 × 10^−08^
Recruitment of NuMA to mitotic centrosomes (R-MMU-380320)	86	11	6.28 × 10^−12^
RAF activation (R-MMU-5673000)	25	3	4.89 × 10^−04^

## Supplementary Materials

The following are available online at https://www.mdpi.com/2218-273X/10/3/355/s1, Supplemental Table S1: Details of mass spectrometry identifications; Supplemental Table S2: PANTHER Cellular Pathways of murine NAGLU^−/−^ brain tissue proteome; Supplemental Table S3: PANTHER GO-Biological Process of murine NAGLU^−/−^ brain tissue proteome; Supplemental Table S4: Protein distribution in Diseases by biomarkers enrichment. Supplemental Figure S1: Reproducibility of the abundance parameter NSAF; Supplemental Figure S2: Normal distribution, Principal Component Analysis (PCA) and correlation matrix for WT and NAGLU^−/−^ protein dataset. Supplemental Figure S3: Volcano plot analysis of global proteome comparison between NAGLU^−/−^ and WT. Supplemental Figure S4: Western blotting of GFAP protein levels in NAGLU^−/−^ and WT brain.
