# Supplementary material for: Proteomic Analysis of Mucopolysaccharidosis IIIB Mouse Brain
Source: Biomolecules. 2020 Feb 26;10(3):355. doi: 10.3390/biom10030355 (PMC7175334; doi:10.3390/biom10030355)
Supplement: Supplementary file 1 [file biomolecules-10-00355-s001.pdf]

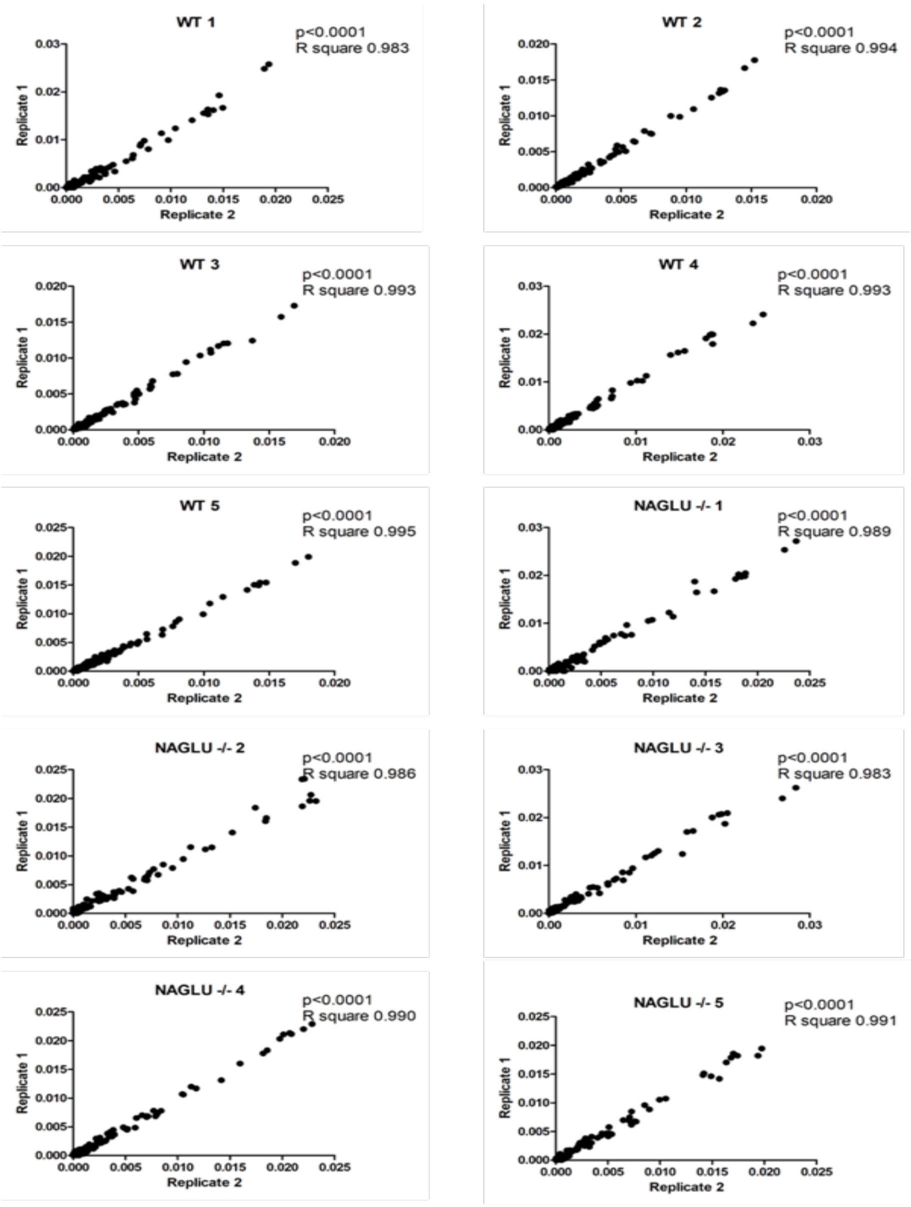

Figure S1. Reproducibility of the abundance parameter NSAF. Linear regression of the correlation between NSAF in the technical replicates. The two axes show the quantitative abundance values, calculated as NSAF of the same proteins, respectively, in the two duplicates from LC-MS/MS analyses. *p-value* and R-squared were evaluated for each linear regression.

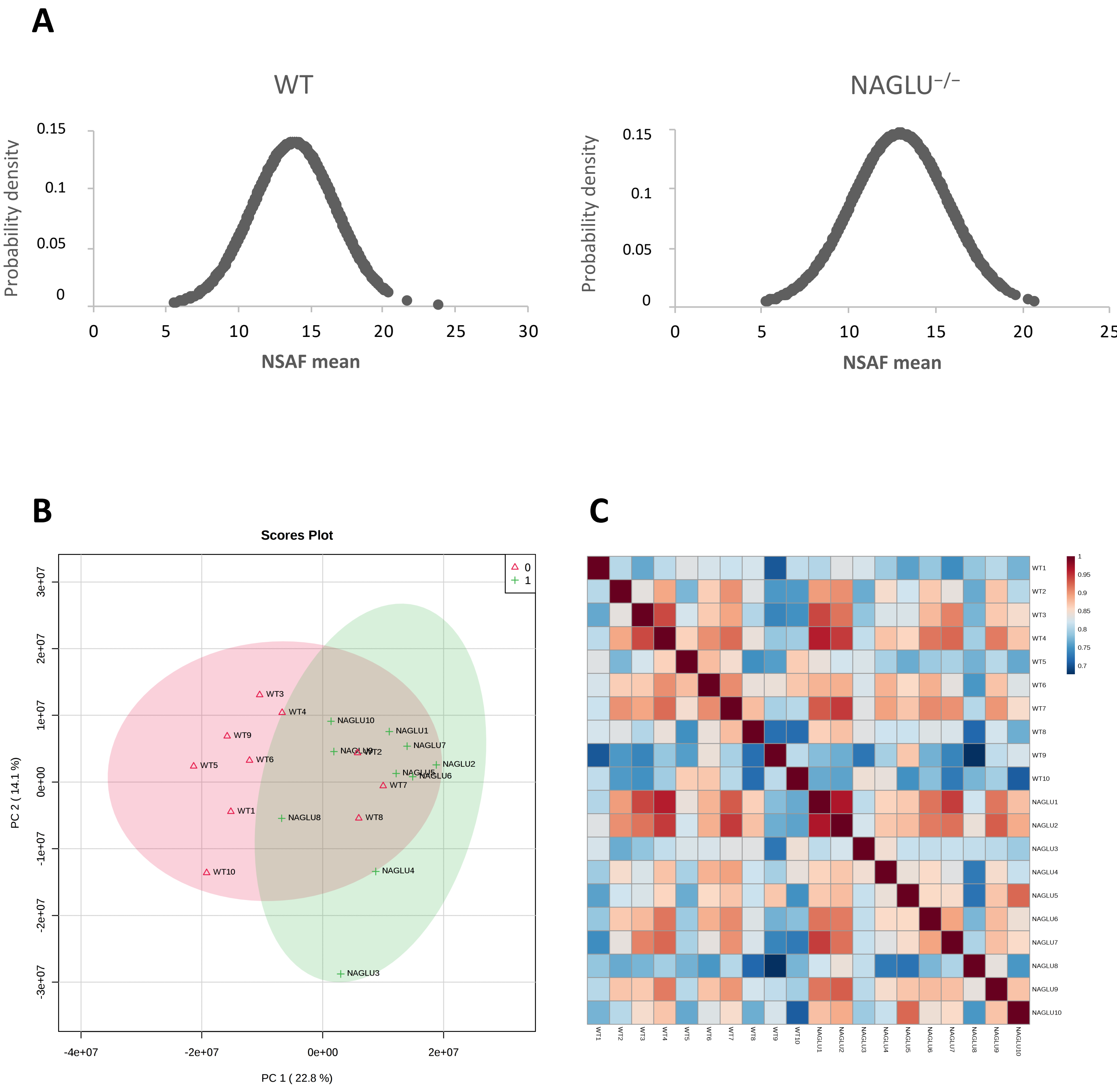

Figure S2. (**A**) Normal distribution of NSAF parameter. The mean of NSAF both in WT and NAGLU$^{-/-}$ dataset was calculated and the log2-transformed NSAF mean was plotted. (**B**) Principal Component Analysis of proteomic data from all brain tissue replicates. WT or NAGLU$^{-/-}$ replicates were represented by a red triangle or green cross, respectively. (**C**) Correlation heatmap. The overall correlation between NAGLU$^{-/-}$ and WT replicates are showed. PCA and Correlation heatmap were performed by Metaboanalyst 4.0.

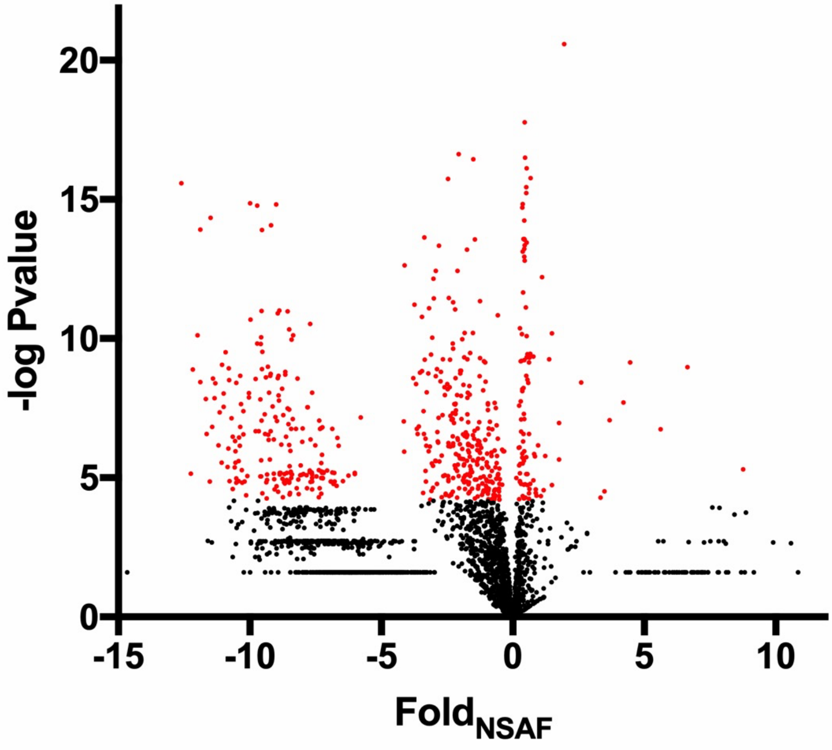

Figure S3. Volcano plot analysis of global proteome comparison between NAGLU$^{-/-}$ and WT. Red circles represent proteins with a significat abundance variantion.

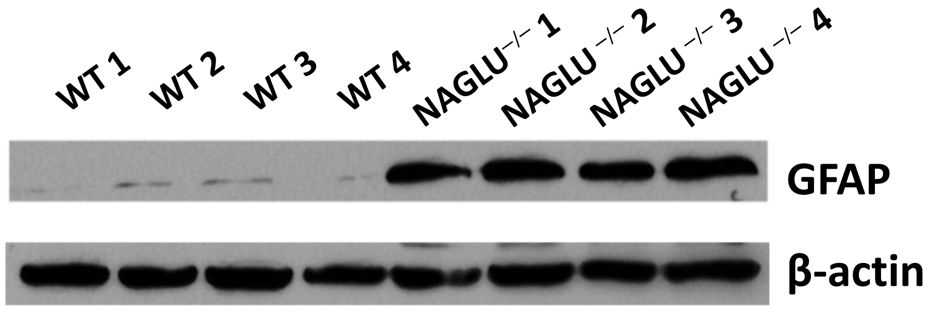

Figure S4. Western blotting of GFAP protein levels in NAGLU$^{-/-}$ and WT brain. Four biological replicates from NAGLU$^{-/-}$ and WT mice were analyzed by using Gfap specific antibody. Equal loading of proteins for each sample was confirmed by beta-actin antibody.
